# FK228 sensitizes radioresistant small cell lung cancer cells to radiation

**DOI:** 10.1186/s13148-021-01025-5

**Published:** 2021-02-25

**Authors:** Hong Li, Liying Ma, Xing Bian, Yang Lv, Wenchu Lin

**Affiliations:** 1 High Magnetic Field Laboratory, Hefei Institutes of Physical Science, Chinese Academy of Sciences, Hefei, 230031 Anhui People’s Republic of China; 2 University of Science and Technology of China, Hefei, 230026 Anhui People’s Republic of China; 3 Key Laboratory of High Magnetic Field and Ion Beam Physical Biology, Hefei Institutes of Physical Science, Chinese Academy of Sciences, Hefei, 230031 Anhui People’s Republic of China

**Keywords:** Small cell lung cancer, FK228, Histone deacetylase, PI3K, Radioresistance, DNA damage repair

## Abstract

**Background:**

Concurrent thoracic radiation plus chemotherapy is the mainstay of first-line treatment for limited-stage small cell lung cancer (LS-SCLC). Despite initial high responsiveness to combined chemo- and radiotherapy, SCLC almost invariably relapses and develops resistance within one year, leading to poor prognosis in patients with LS-SCLC. Developing new chemical agents that increase ionizing radiation’s cytotoxicity against SCLC is urgently needed.

**Results:**

Dual histone deacetylase (HDAC) and PI3K inhibitor FK228 not only displayed potent anticancer activity, but also enhanced the therapeutic effects of radiotherapy in SCLC cells. Mechanistically, radioresistant SCLC cells exhibit a lower level of histone H3K9 acetylation and a higher expression level of the MRE11-RAD50-NBS1 (MRN) complex and show more efficient and redundant DNA damage repair capacities than radiosensitive SCLC cells. FK228 pretreatment resulted in marked induction of H3k9 acetylation, attenuated homologous recombination (HR) repair competency and impaired non-homologous end joining (NHEJ) repair efficacy, leading to the accumulation of radiation-induced DNA damage and radiosensitization.

**Conclusion:**

The study uncovered that FK228 sensitized human radioresistant SCLC cells to radiation mainly through induction of chromatin decondensation and suppression of DNA damage signaling and repair. Our study provides a rational basis for a further clinical study to test the potential of FK228 as a radiosensitizing agent to increase the radiation-induced tumor cell kill in LS-SCLC patients.

## Introduction

Small cell lung cancer (SCLC) is poorly differentiated and high-grade neuroendocrine carcinoma representing about 15–20% of all lung cancer cases [1, 2]. SCLC is classified into limited-stage SCLC (LS-SCLC) and extensive-stage SCLC (ES-SCLC) [3]. LS-SCLC occurs in approximately one-third of patients with SCLC. Despite the high responsiveness to concurrent chest radiation plus chemotherapy, LS-SCLC commonly relapses within months and become resistant to treatment. Radioresistance presents a significant clinical challenge, leading to a high 5-year mortality rate in patients with LS-SCLC [4]. Therefore, further research is needed to dissect the underlying mechanisms of radioresistance and develop new chemical agents to enhance cytotoxicity of ionizing radiation against SCLC cells.

Radiation therapy induces various types of DNA damage, such as base damage, single-strand breaks (SSBs) or double-strand breaks (DSBs), and cross-linking of DNA–DNA. Such DNA insults, if left unrepaired, could eventually result in cell death. A plethora of studies have shown that proficient repair of radiation-induced DNA damage has been strongly associated with radioresistance, whereas defects in specific repair pathways, such as homologous recombination repair, can render the tumors highly sensitive to radiotherapy [5, 6]. Therefore, Targeting DNA damage response (DDR) by small molecules might make SCLC cells more vulnerable to radiotherapy.

The response to DSBs occurs in the context of chromatin. Epigenetic changes in chromatin such as histone modifications and DNA methylation have a significant impact on the DNA repair machinery. Of these, histone acetylation plays a crucial role in DNA damage recognition, signaling, and repair [7, 8]. Indeed, a growing body of data suggests that inhibition of histone deacetylase (HDAC), enzyme regulating cellular acetylation level, impairs the efficiency of DNA damage signaling and the capacity of DNA repair [9, 10]. Consequently, HDACi has been experimentally proven to be able to induce radiosensitization effects on colon, glioma, squamous carcinoma, and many others [11–13]. Furthermore, several clinical trials of HDACi combined with radiation have been carried out and ongoing in a variety of cancers (NCT01010958; NCT01064921; NCT01236560). FK228 (Romidepsin, depsipeptide), a bicyclic tetrapeptide compound with inhibitory activity mainly against class I histone deacetylase enzymes, has been found to have potent anticancer activity and was previously approved by the U.S. Food and Drug Administration (FDA) to treat T cell lymphoma [14, 15]. However, although preclinical studies demonstrated that FK228 showed promising anticancer effects against SCLC [16, 17], a phase II trial of FK228 in sixteen patients with chemosensitive, relapsed SCLC showed limited activity [18]. The precise molecular mechanisms accounting for this limited clinical efficacy remain obscure. A recent preclinical study has demonstrated that FK228 pretreatment augmented IR-triggered apoptosis through induction of BIM expression in gastric cancer cells and squamous carcinoma cells [19, 20]. Whether FK228 acts as a radiosensitizer in SCLC remains still unclear.

In the present study, we found that FK228 inhibited the growth of SCLC cells by inducing apoptosis in vitro. Radioresistant SCLC cells have a higher expression of the MRE11-RAD50-NBS1 (MRN) complex and increased DNA repair capability compared to radiosensitive SCLC cells. FK228 overcame radioresistance in SCLC cells through the modulation of DNA damage signaling and repair. Therefore, our results might offer a potential strategy to improve the therapeutic effects of radiotherapy and reduce radiation resistance in the treatment of LS-SCLC.

## Results

### FK228 inhibited the PI3K signaling pathway and exerted strong cytotoxic effects on SCLC cells

**Fig.1:**
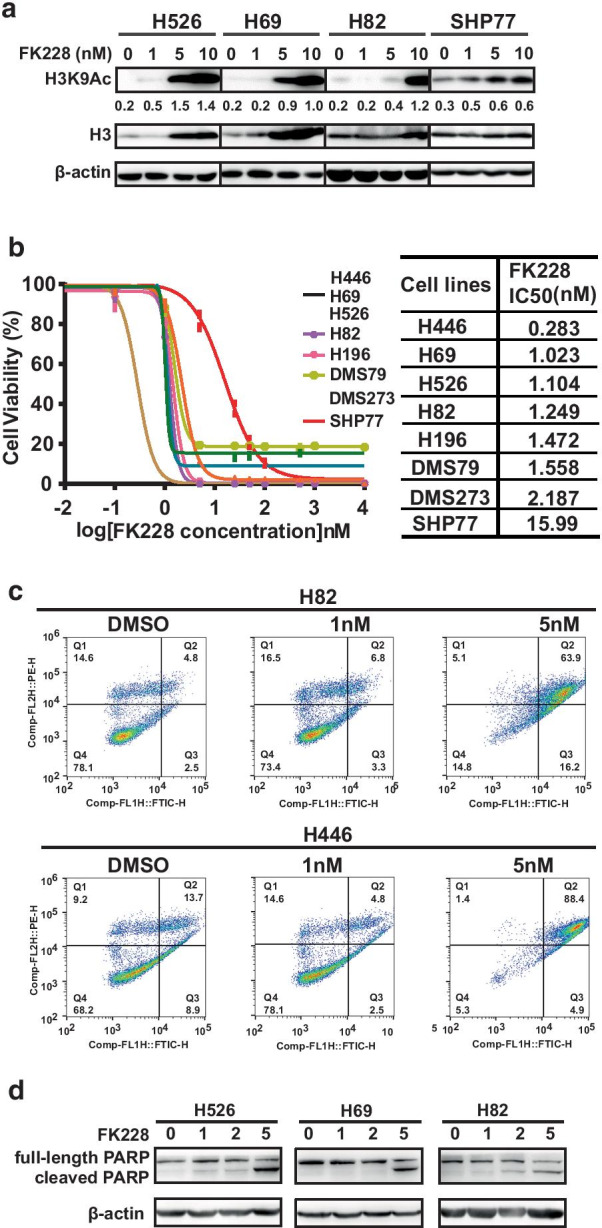
FK228 induces apoptosis and cytotoxicity in a panel of SCLC cell lines. **a** Western blot analysis of acetylated H3 in SCLC cell lines after FK228 for 24 h. **b** Growth inhibition curves of FK228 in a panel of SCLC cell lines. CellTiter-Glo Luminescent assay was performed to evaluate the cell viability after treatment with FK228 for 72 h. The IC50 values were determined from the sigmoidal dose–response curves using PRISM5 software. **c** Annexin V apoptotic assay indicates that FK228 induces apoptosis. SCLC cells were treated with DMSO control, 0.1 nM, 1 nM, and 5 nM FK228 for 48 h. After treatment, Annexin V apoptotic assay was performed by flow cytometry. **d** Western blot analysis of cleaved PARP in three SCLC cell lines after FK228 for 24 h

Previous studies have shown that the PI3K signaling pathway is frequently active in SCLC cells [22], and suppression of the PI3K pathway by histone deacetylase inhibitor (HDACi) is cell type-specific. We began to assess the effect of FK228, a dual HDAC and PI3K inhibitor [23, 24], on the PI3K pathway in SCLC cells. A panel of SCLC cell lines was subjected to 24-h treatment with increased concentrations of FK228. The effects on the PI3K pathway and histone acetylation status were evaluated by western blot analysis. As expected, we observed that the PI3K pathway was modestly inhibited by FK228, as indicated by the dose-dependent decreases in phosphorylation of AKT and its downstream targets, 4EBP-1 and p70S6k in SCLC cell lines examined. The PI3K inhibitory activity was more clearly seen when treated with FK228 at a concentration of 5 nM (Additional files 1, 2). Meanwhile, 24 h of FK228 treatment at nanomolar concentrations led to a dose-dependent accumulation of H3 acetylation (Fig. 1a). To examine the antitumor effects of FK228, a panel of SCLC cells was cultured for 3 days with increased concentrations of FK228, and the growth inhibitory effect of FK228 was measured by CellTiter-Glo assay. All SCLC cell lines examined were sensitive to FK228 treatment with half-maximal inhibitory concentration (IC50) values at low nanomolar ranges (Fig. 1b). However, SHP77 cells were relatively less sensitive to FK228 with IC50 values of over 10 nM than other cell lines. We then carried out a FACS analysis of cell death induced by FK228. Treatment with FK228 dose-dependently induced apoptosis in H82 and H446 cells (Fig. 1c). In accordance with the results of FACS analysis, apoptosis induction by FK228 was supported by a dose-dependent accumulation of PARP cleavage. FK228 at a concentration of 5 nM caused the greatest elevation in the level of PARP cleavage (Fig. 1d). These data indicate that FK228 show antiproliferative and apoptotic effects in SCLC cells in vitro.

### FK228 synergized with radiotherapy in radioresistant SCLC cells

**Fig. 2:**
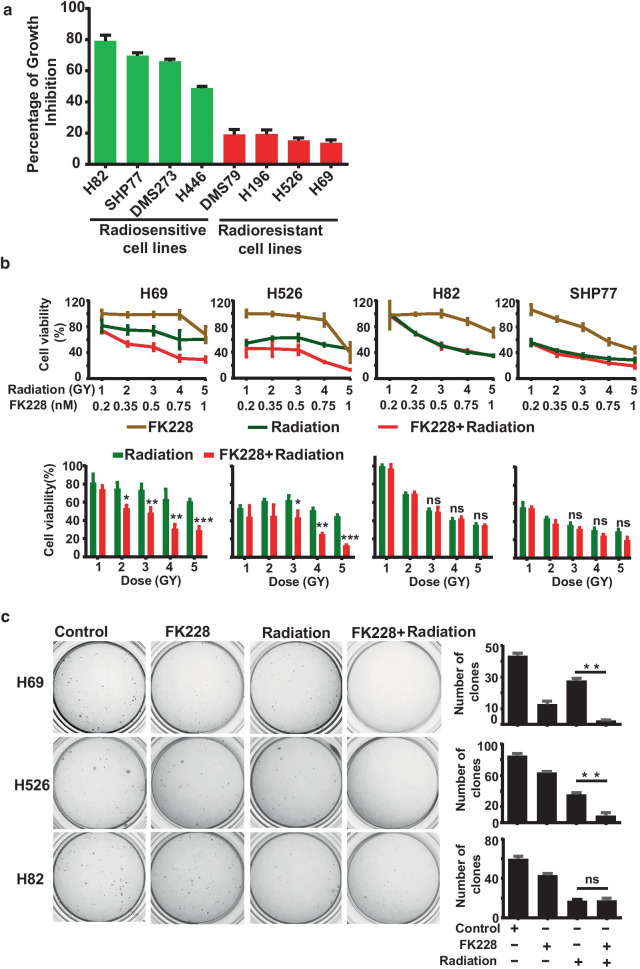
FK228 sensitizes SCLC cells to radiation. **a** The anti-proliferative effect of radiation in a panel of SCLC cell lines. SCLC cell lines were treated with 4 Gy of X-ray irradiation. Growth inhibition was determined by CellTiter-Glo Luminescent assay at 72 h after irradiation. **b** Survival curves (top panel) and survival fractions (bottom panel) of FK228 at different radiation doses. Quantification of survival fraction was shown mean ± SEM. **c** FK228 combined with radiation dramatically suppresses the ability of colony formation in radioresistant SCLC cell lines. Left panels show the representative images of colony formation assay; right panels show the quantification of colony upon treatment with DMSO control, FK228, radiation or the combination. *p < 0.05, **p < 0.01, ***p < 0.001; no, no significance

**Fig. 3:**
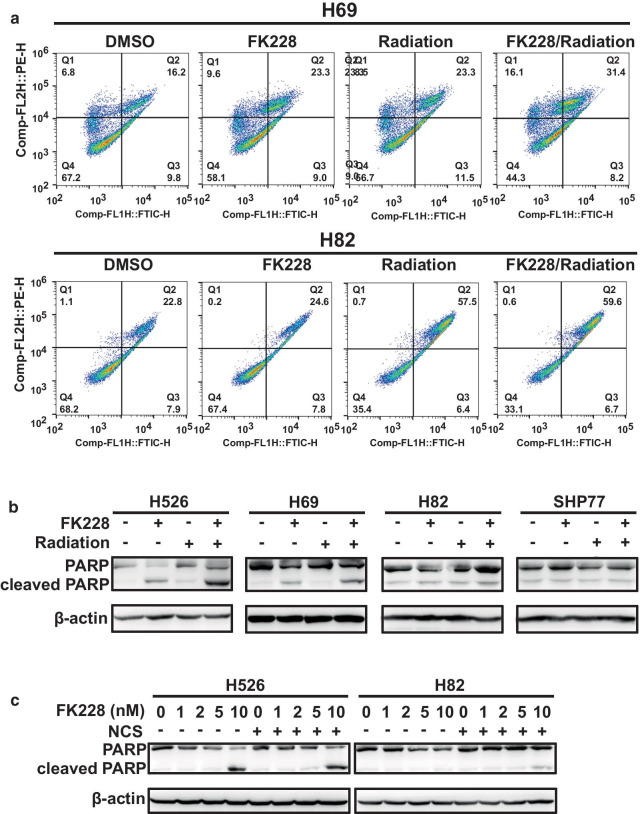
The combination effects of FK228 and radiation. **a** Annexin V apoptotic assay indicates that FK228 enhances radiation-induced apoptosis in radioresistant SCLC cell lines. SCLC cells were treated with DMSO control, 1 nM FK228, radiation or the combination. After treatment, Annexin V apoptotic assay was performed by flow cytometry. **b, c** Western blot analysis of cleaved PARP in SCLC cells upon treatment with FK228 or radiation alone and in combination [30] or FK228 or NCS alone and in combination (**c**)

HDACi is an epigenetic drug that could potentially be used in combination with irradiation [25]. To explore the possibility of FK228 as a radiosensitizer in SCLC, the radiation sensitivity of a panel of SCLC cell lines was characterized. The IC50 values of eight SCLC cell lines following X-ray irradiation were determined using CellTiter-Glo assay. Based on the IC50 difference between the cell lines, the eight cell lines were designated as belonging to either a radiosensitive group consisting of H82, SHP77, DMS273, and H446, or a radioresistant group, comprising of H526, H69, H196, and DMS79 (Fig. 2a). We then examined the effect of combinations of FK228 and X-ray radiation on cell viability in both radioresistant and radiosensitive cells. Strikingly, FK228 treatment enhanced radiation-induced cell death in radioresistant cell lines while there was little to no effect in radiosensitive cell lines (Fig. 2b). We also performed a clonogenic survival assay in three SCLC cell lines, and the results confirmed the radiosensitization by FK228 only in the radioresistant group (Fig. 2c). To further investigate the interaction of the cytotoxic activity of the combination of FK228 with radiation, we also sought to evaluate the contribution of apoptosis by Annexin V staining and measuring the accumulation of PARP cleavage. FACS analysis of cell death demonstrated that pretreatment with FK228 enhanced significantly radiation-induced apoptosis in radioresistant cells. In contrast, no enhanced apoptosis was seen after the combination of FK228 and radiation in radiosensitive cells (Fig. 3a). Furthermore, the key factor involved in cell apoptosis was analyzed by western blotting. The PARP cleavage level was significantly induced after the combined treatment of radiation and FK228 compared to FK228 alone (Fig. 3b). Similar results were also observed after the combined use of FK228 and radiomimetic compound neocarzinostatin (NCS) (Fig. 3c). These results establish that FK228 enhances tumor cell radiosensitivity and apoptosis in radioresistant SCLC cells, but not in radiosensitive SCLC cells in vitro.

### FK228 treatment led to accumulation of histone H3K9 acetylation, phosphorylated histone H2AX (γH2AX) and markedly decreased expression of Rad51 in radioresistant SCLC cells

**Fig. 4:**
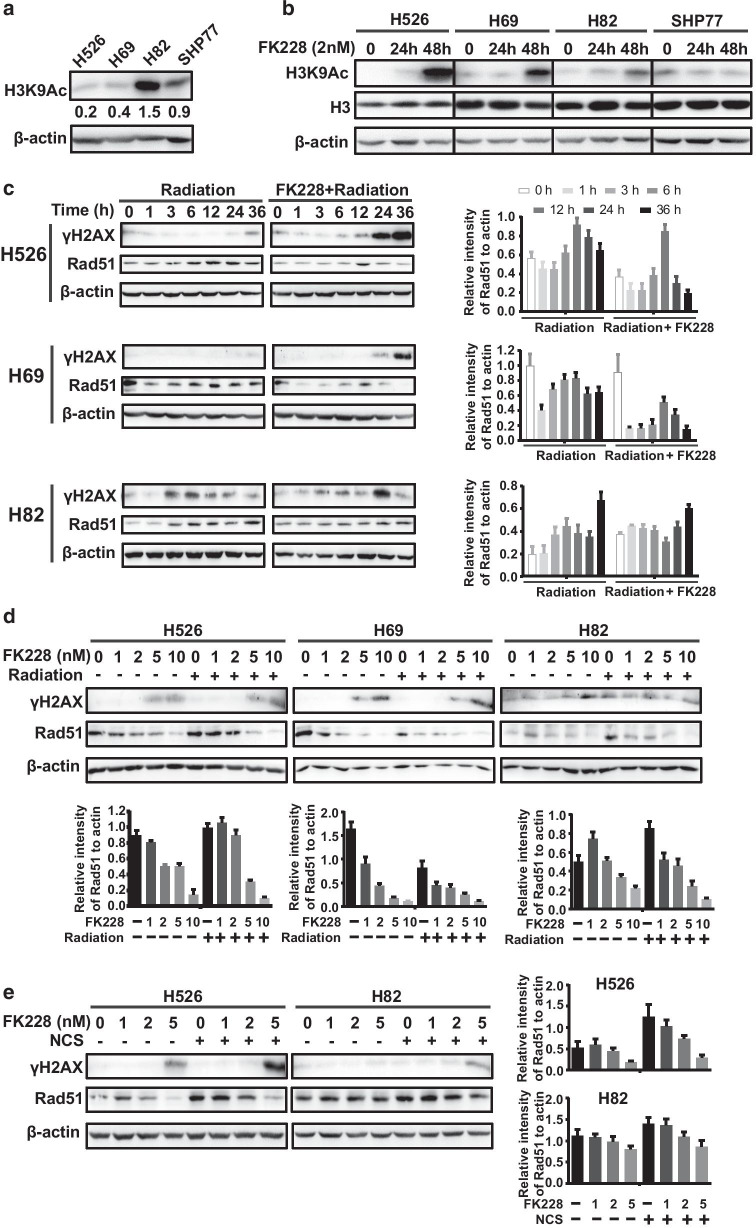
Effects of FK228 and radiation on DDR damage. **a, b** Western blot analysis of the basal level (**a**) and the FK228-induced change (**b**) of histone H3K9Ac in SCLC cell lines. **c** Time-course analysis of γH2AX and Rad51 in SCLC cells by Western blot. SCLC Cells were cultured with 1 nM FK228 for indicated times. **d, e** Dose–response analysis of γH2AX and Rad51 in SCLC Cells treated with radiation (4 Gy) (**d**) or NCS (**e**), 1 nM FK228, or in combination by Western blot. β-actin was used as a loading control. Relative amount of Rad51 were determined by densitometric analysis

A previous report showed that compact chromatin architecture conferred radioresistance [26]. We wondered whether chromatin structure was different between the radioresistant cells and the radiosensitive cells. Western blot analysis showed that the basal levels of histone H3K9 acetylation in H526 and H69 cells were remarkably lower than that in H82 and SHP77 cells (Fig. 4a), indicating a compact chromatin architecture in radioresistant cells. Both dose–response and time-course analysis demonstrated that FK228 resulted in much stronger induction of histone H3K9 acetylation in the radioresistant H526 and H69 cells than the radiosensitive H82 and SHP77 cells (Figs. 1a, 4b). These results indicate that histone acetylation-mediated chromatin change might be responsible for radioresistance.

To further gain insight into the mechanism through which FK228 enhances the radiosensitivity of SCLC cells, DSB repair was evaluated in SCLC cells after drug treatment using phosphorylated histone H2AX (γH2AX) expression assay. Three SCLC cell lines were subjected to treatment with 1 nM of FK228 alone or in combination with 4 G of X-ray irradiation. γH2AX expression was examined at multiple time points after FK228 treatment alone or in combination with irradiation. Time-course experiments revealed that there was a detectable level of accumulation of γH2AX as early as 24 h upon FK228 treatment alone, whereas pretreatment with FK228 significantly increased radiation-induced γH2AX expression in H526 and H69 cells (Fig. 4c). In addition, pretreatment of cells with FK228 induced γH2AX expression in radioresistant cells as a function of dose. It is worth noting, however, there was no accumulation of γH2AX in response to FK228 in radiosensitive H82 cells (Fig. 4d).

HDACi treatment has been found to attenuate the expression of Rad51, a key recombination mediator. We examined the effect of FK228 on Rad51 expression using dose–response and time-course analyses. Time-course experiments showed that the expression of Rad51 was induced at 3 h with peak levels at ≈12 h and declined toward control level by 36 h by FK228 alone (Fig. 4c). When FK228 was combined with irradiation, pretreatment with FK228 blunted Rad51 expression as early as 24 h. Most importantly, the pattern of FK228-induced decrease of Rad51 was seen in radioresistant H526 and H69 cells. In contrast, treatment with FK228 alone had only a slight effect on Rad51 expression in radiosensitive H82 cells (Fig. 4c). Furthermore, radioresistant cells displayed a continuous decrease in Rad51 protein with increasing concentrations of FK228, which was enhanced after the combination treatment (Fig. 4d). Similarly, the patterns of FK228-induced accumulation of γH2AX and decrease in Rad51 protein were seen after combined treatment with FK228 and NCS (Fig. 4e). Together, these results indicate that FK228 alone or in combination with radiation results in a dose and time-dependent accumulation of γH2AX and decreased expression of Rad51 only in radioresistant SCLC cells.

### FK228 induced DNA damage as monotherapy or in combination with radiation

**Fig. 5:**
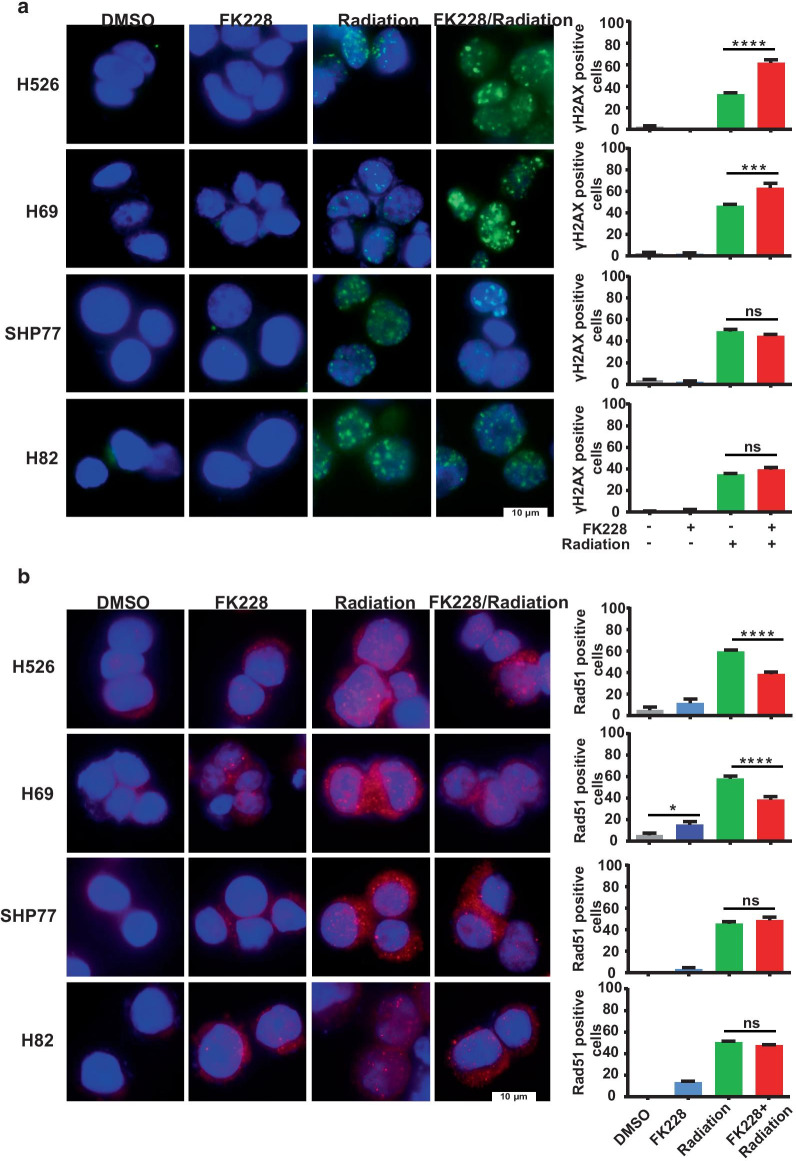
Effects of FK228 and radiation on the DNA double-strand break formation and repair. **a**, **b** Representative images of immunofluorescent staining for γH2AX [31] and Rad51 (30) in SCLC cells upon treatment with FK228 (1 nM) or radiation (4 Gy) alone and in combination. Scale bar, 10 μm. Quantification of γH2AX (**a**) and Rad51 (**b**) fluorescence intensities from three independent experiments was shown in right panels. *p < 0.05, ***p < 0.001, ****p<0.0001; ns, no significance

To further evaluate DNA double-strand break (DSB) formation and repair, Immunofluorescence staining was performed to measure the formation of γH2AX and Rad51 foci. As shown in Fig. 5a, FK228 augmented formation of radiation-induced γH2AX foci, whereas FK228 alone induced little to no formation of γH2AX in H526 and H69 cells. Conversely, the addition of FK228 had little or no effect on the radiation-dependent γH2AX focus formation in H82 and SHP77 cells. In support of the results of γH2AX foci analysis, immunofluorescence staining for Rad51 revealed jeopardized formation of Rad51 foci in H526 and H69 cells treated with the combination of FK228 and radiation, whereas there was no difference in the radiation-induced formation of Rad51 foci in H82 and SHP77 cells upon FK228 treatment compared to radiation alone (Fig. 5b). These data indicate that FK228 impairs the recruitment of Rad51 to the sites of DSBs and augments radiation-induced DNA damage in radioresistant SCLC cells.

### Radioresistant SCLC cells had a higher expression of the MRE11-RAD50-NBS1 (MRN) complex and exhibited more potent DSB repair activity

**Fig. 6:**
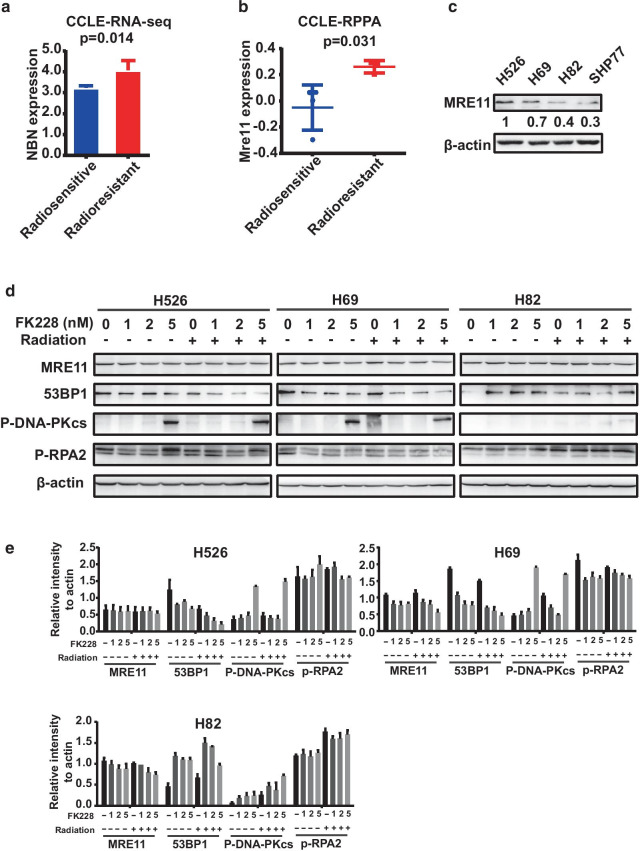
MRN complex affects DDR response to FK228 and radiation. **a** Relative expression of *NBN* in radioresistant SCLC cell lines compared to radiosensitive cell lines. **b** Relative expression of MRE11 expression at protein level in radioresistant SCLC cell lines compared to radiosensitive cell lines. **c** Western blot analysis of MRE11 showing that radioresistant SCLC cell lines have more MRE11 expression than radiosensitive cells. **d** Western blot analysis of MRE11, 53BP1, P-DNA-PKcs, P-RPA2 in SCLC Cells treated with radiation (4 Gy) (**b**), 1 nM FK228, or in combination. β-actin was used as a loading control. **e** Relative amount of proteins in SCLC Cells treated with radiation (4 Gy), 1 nM FK228, or in combination were determined by densitometric analysis. The quantification data presented were the average densitometric value of three independent western blotting experiments

Radioresistance is the main impediment to effective radiotherapy for SCLC. Despite recent advances in radiotherapy, the precise mechanisms of radioresistance in SCLC cells and tissues have yet to be fully understood. Given that DSB is the major radio-toxic damage and the effects of DSBs induce by FK228 were different between the radioresistant SCLC cells and the radiosensitive SCLC cells, we sought to investigate the role of the DDR pathway in radiosensitivity in SCLC. We first analyzed the CCLE RNA-seq data set for the expression of DDR genes. The expression of *NBN* that encodes a core component of the MRN complex in radioresistant cells was significantly higher than that in radiosensitive cells (Fig. 6a). In addition, *RAD50* and *RAD51* tend to be highly expressed in radioresistant SCLC cells (data not shown). Moreover, evaluation of the Reverse Phase Protein Array (RPPA) data set from the Cancer Cell Line Encyclopedia (CCLE) showed that MRE11, a component of the MRN complex, was highly expressed at the protein level in radioresistant SCLC cells (Fig. 6b). The high expression of Rad51 at the protein level was also associated with radioresistant cells even though a p-value did not reach statistical significance (data not shown). Furthermore, western blotting analysis demonstrated that the baseline levels of MRE11 were significantly higher in H526 and H69 cells than in H82 cells (Fig. 6c). These results indicate that the radioresistant SCLC cells have a higher expression of MRX complex and a possible higher competency in HR and NHEJ than the radiosensitive SCLC cells.

### *FK228 sensitized SCLC cells to radiotherapy *via* attenuation of DSB repair capacity*

The MRN complex sits at a central position in the DDR network in response to DSBs, dictating the pathway choice in DSB repair. Based on the correlation between the high expression of the MRN complex component and radioresistance, we wondered whether MRE11 expression confers different effects under distinct contexts. To further dissect the molecular mechanism underlying the radiosensitivity of FK228 observed in SCLC cells, we investigated the effects of FK228 alone or in combination with radiation on the DDR pathway. Both radioresistant and radiosensitive cells were treated with 1, 2, or 5 nM FK228 alone or in combination with radiation for 24 h and then subjected to western blotting analysis. The phosphorylation of RPA32, a DNA end-resection marker, was down-regulated in H526 and H69 cells. We also examined the effects of FK228 on the expression of HR and NHEJ genes. FK228 treatment did cause a dose-dependent decrease of Rad51 and 53BP1 in radioresistant but not in radiosensitive cells (Figs. 4d, 6d, e). Furthermore, there was an accumulation of phospho-DNA-PKcs that occurred in radioresistant but not in radiosensitive cells (Fig. 6d, e). Overall, these data indicate that the radiosensitization achieved by FK228 could be attributed to its inhibitory effects on both HR and NHEJ repair.

## Discussion

Romidepsin was approved by the FDA for use in treating refractory cutaneous and peripheral T cell lymphoma [14, 15]. However, Clinical trials of FK228 in several solid tumors, including SCLC, have shown limited success. The factors that limit the efficacy of FK228 in solid tumors remain still unclear. In the present study, we showed that FK228, a dual HDAC and PI3K inhibitor identified recently, displayed similar inhibitory activity to BEZ235 on PI3K pathway (Additional file 2) and exhibited potent cytotoxic effects by inducing apoptosis in the investigated SCLC cell lines. More importantly, we discovered that FK228 was more effective in enhancing radiosensitivity in radioresistant cells than that in radiosensitive cells. FK228 could act as a radiosensitizer in a particular setting in SCLC. The mechanistic investigation demonstrated that radioresistant SCLC cell had a more efficient capacity to repair the DNA DSBs compared with radiosensitive SCLC cell as evidenced by higher expression of MRN complex components and DSB repair genes. FK228 improved the treatment efficacy of radiation in SCLC cells through the impairment of HR and NHEJ repair.

Although radiotherapy is widely used in clinical applications against SCLC, numerous patients are prone to relapse due to the intrinsic or adaptive resistance of cancer cells to radiation. A better understanding of the underlying mechanisms of radioresistance could significantly extend its applications. Deficiencies in DSB repair pathways are believed to be related to radiosensitivity. Our investigation confirmed that the difference of cellular DNA repair capacities between cell lines might explain the different behaviors to radiation. The cells having competent DSB repair activity can have more power to fix radiation-induced DSBs, therefore resulting in radioresistance. Our results are in agreement with several studies in which targeting either MRN complex or RAD51 sensitize tumor cells to radiation [5, 27, 28]. Therefore, identification of defects in DSB repair pathways in patients with SCLC offers a potential therapeutic avenue to inform treatment of SCLC. Future characterization of the sensitizing effect of FK228 and potential biomarkers for the combined use of FK228 and radiotherapy in vivo will further illuminate the diverse antitumor and radiosensitizing effects of FK228.

Numerous lines of evidence have shown that HDACi is an excellent candidate to be used as a radiosensitizing agent. Although Pan-HDAC inhibitors such as panobinostat and vorinostat exert high activity as radiosensitizers, more selective HDAC inhibitors might be better sensitizer to radiotherapy since its cause fewer side effects. In our study, we found that FK228, a class I HDAC inhibitor, radiosensitized SCLC cells. A previous report showed that HDACi radiosensitized human thyroid cancer cell lines but not the normal thyroid follicular epithelial cells [29]. In line with the selectivity of HDACi for radiosensitizing cancer cells, we observed that FK228 sensitized radioresistant SCLC cells to radiation. Similar selectivity of HDACi for killing cancer cells was also reported in human prostate and lung cancer cells, in which normal but not cancer cells repair DSBs [10]. The mechanisms that cause different effects of FK228 on the DNA repair machinery in SCLC cells remain to be defined.

## Conclusion

Our report provides in vitro evidence that FK228 radiosensitizes human SCLC cells mainly by inhibiting the DNA repair machinery. Our preclinical data presented here suggest that radioresistant SCLC might benefit from FK228, especially in concert with radiation. These findings might have a significant clinical implication for the management of patients with LS-SCLC who have a high risk of recurrence and fail to respond to radiotherapy.

## Materials and methods

### Reagents and antibodies

FK228 was purchased from Selleck Chemical (Shanghai, China) and neocarzinostatin (NCS) was purchased from Sigma-Aldrich (Saint Louis, MO, USA). Stock solutions prepared in dimethyl sulfoxide (DMSO) (Sigma-Aldrich, Saint Louis, MO, USA) were stored at − 20 °C and diluted to the final concentration in media before each experiment. Antibodies against phosphorylated histone H2AX, PARP, Phospho-Akt (Ser473), Akt, Phospho-4E-BP1(Ser65), 4E-BP1, Phospho-S6 Ribosomal Protein (Ser235/236), S6 Ribosomal Protein, acetyl-Histone H3 (lys9), histone H3 were purchased from Cell Signaling Technology (Danvers, MA, USA). Antibodies against Rad51, MRE11, and p-DNA-PKcs (S2056) were from Abcam (London, United Kingdom). The acetyl-Ku70 (K539) antibody was from ImmunoWay Biotech (Plano, TX, USA). Ku70 antibody was from Affinity Biosciences (Cincinnati, OH, USA). The p-RPA2 (Ser4/Ser8) antibody was from Novus Biologicals (Centennial, CO, USA). β-Actin antibody was from TransBionovo (Beijing, China).

### Cell culture

Human small cell lung cancer (SCLC) cell lines were grown routinely in RPMI1640 medium (Cellgro, Manassas, VA, USA) containing 10% fetal bovine serum (BioInd, Israel) and 1% penicillin/streptomycin (Gibco, Life Technologies, Carlsbad, CA, USA). Cells were kept at 37 °C in a humidified atmosphere of 5% CO_2_ and 95% filtered air. Exponential growing cells were used for the experiments.

### X-ray irradiation

SCLC cells were irradiated at varying doses in X-RAD 320 Biological Irradiator (Precision X-Ray, North Branford, CT, USA). For the irradiation/FK228 combination treatment group, radiation was administered to the cells after 16 h of FK228 exposure.

### Cell viability assay

SCLC cells were seeded in triplicates in 96-well plates at a density of 1000 cells per well and the cell viability was detected by the CellTiter-Glo luminescent assay according to the manufacturer’s instructions after 72 h of drugs treatment. The luminescence signal was recorded with a multilabel plate reader (Envision PerkinElmer, USA).

### Colony formation assay

To evaluate colony-forming potential after drug treatment, SCLC cells were seeded into 60 mm dishes in triplicates for each condition and incubated for 24 h. Cells were then treated with DMSO control, FK228, radiation, and radiation/FK228 combination (FK228 pretreatment for 16 h, followed by 4 Gy after treatment). 2 × 10^3^ cells in 1 ml of RPMI1640 containing 10% (v/v) FBS and 0.33% (w/v) agarose were overlaid onto bottom agar consisting of 1 ml of RPMI1640 containing 10% (v/v) FBS and 0.5% (w/v) agarose in 6-well plates. The cells were then incubated at 37 °C in 5% CO2 for 10 to 20 days to allow colony formation. Colonies were photographed, and colonies containing > 50 cells were counted for each condition and normalized to the plating efficiency of each line.

### Cell apoptosis assay

The SCLC cells were plated in 6-well plates and allowed to grow overnight. The cells were then treated with DMSO control, FK228, radiation, and radiation/FK228 combination in the second day for 48 h. The cell apoptotic rate was determined by flow cytometry using a FITC Annexin V Apoptosis Detection kit (BD Pharmingen, San Diago, CA, USA) according to the manufacturer’s instructions. The samples were then analyzed by FACS Calibur (BD Pharmingen, San Diago, CA, USA).

### Immunofluorescence staining

3 h after irradiation cells were fixed with 4% paraformaldehyde containing 2% sucrose for 10 min and then washed with PBS. Cells were then permeabilized with 0.25% Triton X-100 for 10 min, blocked with 1% BSA for 1 h, and incubated with anti-gamma H2A.X (phospho S139) antibody (1:500, CST, #2577), anti-Rad51 antibody (1:500, Abcam, ab133534) in PBS containing 1% BSA overnight at 4 °C. Cells were then washed with PBS and incubated with FITC-conjugated secondary antibody for 2 h at room temperature. Cells were finally washed with PBST and then stained for 5 min with DAPI. The images were captured by a Zeiss fluorescent microscope (Olympus, Japan). The image analysis was done by using Image J (National Institutes of Health). At least 100 cells were counted for each condition, and cells with more than 5 foci were considered positive.

### Western blotting

Cells were lysed as described previously [21], and total protein was quantified by BCA Protein Assay Kit (Beyotime, Shanghai, China). Proteins were then separated by SDS-PAGE, transferred onto nitrocellulose blotting membrane (Cell Signaling Technology, Danvers, MA, USA). The membranes were washed and incubated with a primary antibody at 4 °C overnight, followed by incubation with a secondary antibody for 2 h at room temperature. Signals were visualized using SuperSignal West Pico chemiluminescent substrate (Thermo Scientific, Rockford, IL, USA).

### SCLC cell line data processing and analysis

RNA-seq data from 50 SCLC cell lines and reverse-phase protein array (RPPA) data from 45 SCLC cell lines, and general information for these cell lines, were downloaded from https://portals.broadinstitute.org/ccle/data. Expression data for DDR genes were extracted, analyzed, and displayed in scatter plots.

### Statistical analysis

All in vitro analyses were repeated at least in triplicate. Statistical analysis was performed using GraphPad Prism software. Quantitative results were analyzed using two-tailed unpaired Student’s *t*-tests. *p *Values < 0.05 were considered statistically significant.

## Supplementary Information

**Additional file 1**. Western blot analysis of phospho-proteins downstream of PI3K signaling in SCLC cell lines after FK228 for 24 h.

**Additional file 2**. **a** Western blot analysis of phospho-proteins downstream of PI3K signaling in SCLC cell lines following 24-hour drug treatment. BEZ235, 100 nM; SAHA, 1 mM; FK228, 10 nM. **b** Western blot analysis showing the change of acetylated Ku70 in SCLC cell lines after FK228 for 24 h.

## Data Availability

Research data are stored in an institutional repository and will be shared upon request to the corresponding author.

## Abbreviations

SCLC: Small cell lung cancer
LS-SCLC: Limited-stage small cell lung cancer
ES-SCLC: Extensive-stage small cell lung cancer
HR: Homologous recombination
NHEJ: Non-homologous end joining
SSBs: Single-strand breaks
DSBs: Double-strand breaks
DDR: DNA damage response
HDAC: Histone deacetylase
HDACi: Histone deacetylase inhibitor
FDA: U.S. Food and Drug Administration
NCS: Neocarzinostatin
IC50: Half-maximal inhibitory concentration
FACS: Fluorescence Activated Cell Sorting
H3K9: Histone H3 lysine 9
γH2AX: Phosphorylated histone H2AX
RPPA: Reverse Phase Protein Array
CCLE: Cancer Cell Line Encyclopedia
MRN: MRE11-RAD50-NBS1
