# Supplementary figures and images for: FK228 sensitizes radioresistant small cell lung cancer cells to radiation
Source: Clin Epigenetics. 2021 Feb 25;13:41. doi: 10.1186/s13148-021-01025-5 (PMC7905898; doi:10.1186/s13148-021-01025-5)

Additional file 1

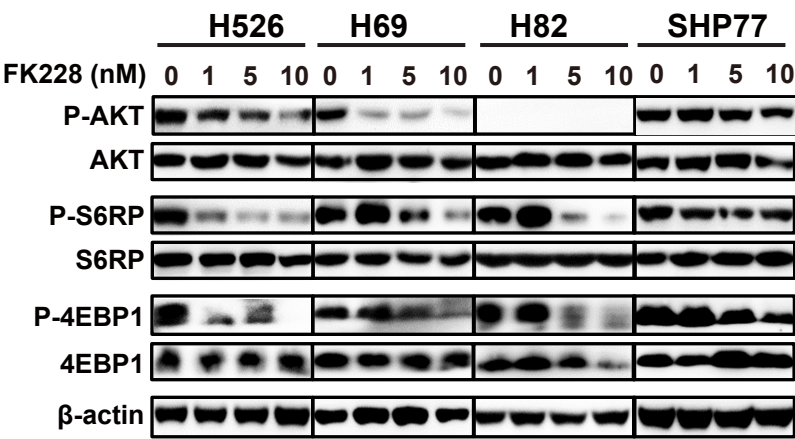

Supplement: Supplementary file 1 — Additional file 1. Western blot analysis of phospho-proteins downstream of PI3K signaling in SCLC cell lines after FK228 for 24 h. [file 13148_2021_1025_MOESM1_ESM.pdf]

Additional file 2

a

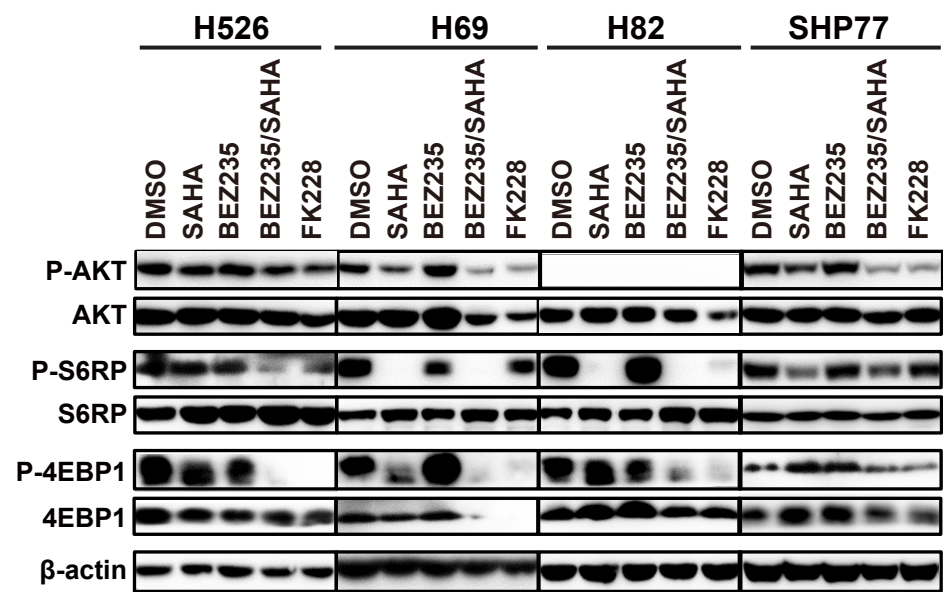

b

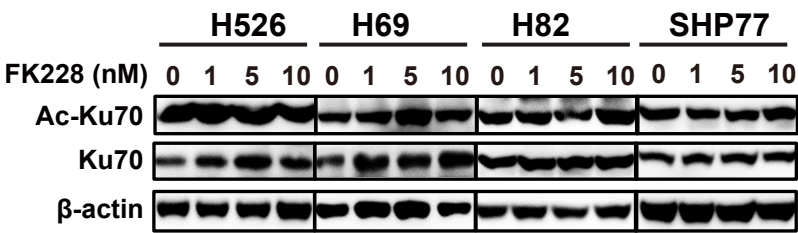

Supplement: Supplementary file 2 — Additional file 2. a Western blot analysis of phospho-proteins downstream of PI3K signaling in SCLC cell lines following 24-hour drug treatment. BEZ235, 100 nM; SAHA, 1 mM; FK228, 10 nM. b Western blot analysis showing the change of acetylated Ku70 in SCLC cell lines after FK228 for 24 h. [file 13148_2021_1025_MOESM2_ESM.pdf]
